# Computing strategies for multi-population genomic evaluation

**DOI:** 10.1186/s12711-022-00705-x

**Published:** 2022-02-04

**Authors:** Andrés Legarra, David González-Diéguez, Zulma G. Vitezica

**Affiliations:** 1 INRAE, INP, UMR 1388 GenPhySE, 31326 Castanet-Tolosan, France; 2 International Maize and Wheat Improvement Center (CIMMYT), Apdo. Postal 6-641, 06600 Mexico, Mexico

## Abstract

**Background:**

Multiple breed evaluation using genomic prediction includes the use of data from multiple populations, or from parental breeds and crosses, and is expected to lead to better genomic predictions. Increased complexity comes from the need to fit non-additive effects such as dominance and/or genotype-by-environment interactions. In these models, marker effects (and breeding values) are modelled as correlated between breeds, which leads to multiple trait formulations that are based either on markers [single nucleotide polymorphism best linear unbiased prediction (SNP-BLUP)] or on individuals [genomic(G)BLUP)]. As an alternative, we propose the use of generalized least squares (GLS) followed by backsolving of marker effects using selection index (SI) theory.

**Results:**

All investigated options have advantages and inconveniences. The SNP-BLUP yields marker effects directly, which are useful for indirect prediction and for planned matings, but is very large in number of equations and is structured in dense and sparse blocks that do not allow for simple solving. GBLUP uses a multiple trait formulation and is very general, but results in many equations that are not used, which increase memory needs, and is also structured in dense and sparse blocks. An alternative formulation of GBLUP is more compact but requires tailored programming. The alternative of solving by GLS + SI is the least consuming, both in number of operations and in memory, and it uses only single dense blocks. However, it requires dedicated programming. Computational complexity problems are exacerbated when more than additive effects are fitted, e.g. dominance effects or genotype x environment interactions.

**Conclusions:**

As multi-breed predictions become more frequent and non-additive effects are more often included, standard equations for genomic prediction based on Henderson’s mixed model equations become less practical and may need to be replaced by more efficient (although less general) approaches such as the GLS + SI approach proposed here.

## Background

For genomic prediction in livestock and crops, marker effects are often modelled as different but correlated effects across populations [1, 2]. This results in a multiple trait setting, in which each environment or population is modelled as a different trait. As individuals are present in a single environment, there is no covariance between other random effects (such as residual or permanent environmental effects). This leads to particular structures of the incidence matrices that make general computational strategies less efficient. In this work, we discuss some of these strategies, show that general strategies such as standard individual-based multiple trait genomic best linear unbiased prediction (GBLUP) leads to high computational redundancy, and we highlight that the old method of generalized least squares (GLS) followed by selection index (SI) [3, 4] is a competing strategy in terms of efficiency. The motivation for the comparison of these strategies was the need to obtain estimates of marker effects in a computationally efficient manner, for their use in planning assortative matings in a two-way breeding scheme [5], using data from two purebred populations and a crossbred population, for a total of ~ 50K animals and genotypes at ~ 50K single nucleotide polymorphisms (SNPs). Here, we are concerned with medium-sized data sets, but with very complex models, where genetic evaluation can be done by exact (non-iterative) methods, i.e. by numerical inversion. This is a popular strategy for genomic evaluation in crops and in some populations of monogastric livestock.

## Methods

For our argumentation, and following the example of [5], we assume additive ($a$) and dominant ($d$) SNP effects that are correlated across the different populations. In principle, the model can be extended to higher-order effects such as epistasis. The dominance effects have an a priori mean of 0 because genomic inbreeding is included in the model as a covariate [6]. For the example, we consider three populations but any number of populations can be accommodated. For each population $i$ $(i=\left\{\mathrm{1,2},3\right\})$, we have a single trait vector of phenotypes, ${\mathbf{y}}_{i}$, with the following linear model:

$${\mathbf{y}}_{i}={\mathbf{X}}_{i}{{\varvec{\upbeta}}}_{i}+{\mathbf{Z}}_{i}{\mathbf{a}}_{i}+{\mathbf{W}}_{i}{\mathbf{d}}_{i}+{\mathbf{e}}_{i},$$

where ${{\varvec{\upbeta}}}_{i}$ contains the fixed effects specific to population $i$ (e.g., a mean and an inbreeding depression covariate), ${\mathbf{a}}_{i}$ and ${\mathbf{d}}_{i}$ are additive and dominance SNP effects, respectively, specific to population $i$, with incidence matrices coded, e.g., as ${\mathbf{Z}}_{i}=\left\{-\mathrm{1,0},1\right\}$ and ${\mathbf{W}}_{i}=\{\mathrm{0,1},0\}$. Other codings are possible, such as in terms of breeding values and dominance deviations to achieve orthogonality [7]. The covariance structure, written using the Kronecker product ⨂, is:

$$Var\left(\begin{array}{c}{\mathbf{a}}_{1}\\ {\mathbf{a}}_{2}\\ {\mathbf{a}}_{3}\end{array}\right)={\mathbf{G}}_{0a}\otimes \mathbf{I}=\left(\begin{array}{ccc}{\mathbf{I}\sigma }_{a1}^{2}& \mathbf{I}{\sigma }_{a12}& \mathbf{I}{\sigma }_{a13}\\ \mathbf{I}{\sigma }_{a21}& {\mathbf{I}\sigma }_{a2}^{2}& \mathbf{I}{\sigma }_{a23}\\ \mathbf{I}{\sigma }_{a31}& \mathbf{I}{\sigma }_{a32}& {\mathbf{I}\sigma }_{a3}^{2}\end{array}\right),$$

for $${\mathbf{G}}_{0a}=\left(\begin{array}{ccc}{\sigma }_{a1}^{2}\boldsymbol{ }& {\sigma }_{a12}& {\sigma }_{a13}\\ {\sigma }_{a21}& {\sigma }_{a2}^{2}& {\sigma }_{a23}\\ {\sigma }_{a31}& {\sigma }_{a32}& {\sigma }_{a3}^{2}\end{array}\right)$$, the covariance matrix of additive marker effects, with inverse $${\mathbf{G}}_{0a}^{-1}=\left(\begin{array}{ccc}{g}_{0a}^{11}\boldsymbol{ }\boldsymbol{ }& {g}_{0a}^{12}& {g}_{0a}^{13}\\ {g}_{0a}^{21}& {g}_{0a}^{22}& {g}_{0a}^{23}\\ {g}_{0a}^{31}& {g}_{0a}^{32}& {g}_{0a}^{33}\end{array}\right)$$.

$$Var\left(\begin{array}{c}{\mathbf{d}}_{1}\\ {\mathbf{d}}_{2}\\ {\mathbf{d}}_{3}\end{array}\right)={\mathbf{G}}_{0d}\otimes \mathbf{I}=\left(\begin{array}{ccc}{\mathbf{I}\sigma }_{d1}^{2}& \mathbf{I}{\sigma }_{d12}& \mathbf{I}{\sigma }_{d13}\\ \mathbf{I}{\sigma }_{d21}& {\mathbf{I}\sigma }_{d2}^{2}& \mathbf{I}{\sigma }_{d23}\\ \mathbf{I}{\sigma }_{d31}& \mathbf{I}{\sigma }_{d32}& {\mathbf{I}\sigma }_{d3}^{2}\end{array}\right),$$

for $${\mathbf{G}}_{0d}=\left(\begin{array}{ccc}{\sigma }_{d1}^{2}\boldsymbol{ }& {\sigma }_{d12}& {\sigma }_{d13}\\ {\sigma }_{d21}& {\sigma }_{d2}^{2}& {\sigma }_{d23}\\ {\sigma }_{d31}& {\sigma }_{d32}& {\sigma }_{d3}^{2}\end{array}\right)$$, with inverse $${\mathbf{G}}_{0d}^{-1}=\left(\begin{array}{ccc}{g}_{0d}^{11}\boldsymbol{ }\boldsymbol{ }& {g}_{0d}^{12}& {g}_{0d}^{13}\\ {g}_{0d}^{21}& {g}_{0d}^{22}& {g}_{0d}^{23}\\ {g}_{0d}^{31}& {g}_{0d}^{32}& {g}_{0d}^{33}\end{array}\right)$$.

$$Var\left(\begin{array}{c}{\mathbf{e}}_{1}\\ {\mathbf{e}}_{2}\\ {\mathbf{e}}_{3}\end{array}\right)={\mathbf{R}}=\left(\begin{array}{ccc}{\mathbf{I}\sigma }_{e1}^{2}& \mathbf{0}& \mathbf{0}\\ \mathbf{0}& {\mathbf{I}\sigma }_{e2}^{2}& \mathbf{0}\\ \mathbf{0}& \mathbf{0}& {\mathbf{I}\sigma }_{e3}^{2}\end{array}\right),$$

$$\begin{aligned}Var\left(\begin{array}{c}{\mathbf{y}}_{1}\\ {\mathbf{y}}_{2}\\ {\mathbf{y}}_{3}\end{array}\right) & =\mathbf{V}=\left(\begin{array}{ccc}{{\mathbf{Z}}_{1}\sigma }_{a1}^{2}{\mathbf{Z}}_{1}^{\boldsymbol{^{\prime}}}\boldsymbol{ }& {\mathbf{Z}}_{1}{\sigma }_{a12}{\mathbf{Z}}_{2}^{\boldsymbol{^{\prime}}}& {\mathbf{Z}}_{1}{\sigma }_{a13}{\mathbf{Z}}_{3}^{\boldsymbol{^{\prime}}}\\ {\mathbf{Z}}_{2}{\sigma }_{a21}{\mathbf{Z}}_{1}^{\boldsymbol{^{\prime}}}& {{\mathbf{Z}}_{2}\sigma }_{a2}^{2}{\mathbf{Z}}_{2}^{\boldsymbol{^{\prime}}}& {\mathbf{Z}}_{2}{\sigma }_{a23}{\mathbf{Z}}_{3}^{\boldsymbol{^{\prime}}}\\ {\mathbf{Z}}_{3}{\sigma }_{a31}{\mathbf{Z}}_{1}^{\boldsymbol{^{\prime}}}& {\mathbf{Z}}_{3}{\sigma }_{a32}{\mathbf{Z}}_{2}^{\boldsymbol{^{\prime}}}& {{\mathbf{Z}}_{3}\sigma }_{a3}^{2}{\mathbf{Z}}_{3}^{\boldsymbol{^{\prime}}}\end{array}\right)+\left(\begin{array}{ccc}{{\mathbf{W}}_{1}\sigma }_{d1}^{2}{\mathbf{W}}_{1}^{\boldsymbol{^{\prime}}}\boldsymbol{ }& {\mathbf{W}}_{1}{\sigma }_{d12}{\mathbf{W}}_{2}^{\boldsymbol{^{\prime}}}& {\mathbf{W}}_{1}{\sigma }_{d13}{\mathbf{W}}_{3}^{\boldsymbol{^{\prime}}}\\ {\mathbf{W}}_{2}{\sigma }_{d21}{\mathbf{W}}_{1}^{\boldsymbol{^{\prime}}}& {{\mathbf{W}}_{2}\sigma }_{d2}^{2}{\mathbf{W}}_{2}^{\boldsymbol{^{\prime}}}& {\mathbf{W}}_{2}{\sigma }_{d23}{\mathbf{W}}_{3}^{\boldsymbol{^{\prime}}}\\ {\mathbf{W}}_{3}{\sigma }_{d31}{\mathbf{W}}_{1}^{\boldsymbol{^{\prime}}}& {\mathbf{W}}_{3}{\sigma }_{d32}{\mathbf{W}}_{2}^{\boldsymbol{^{\prime}}}& {{\mathbf{W}}_{3}\sigma }_{d3}^{2}{\mathbf{W}}_{3}^{\boldsymbol{^{\prime}}}\end{array}\right)+\left(\begin{array}{ccc}{\mathbf{I}\sigma }_{e1}^{2}\boldsymbol{ }& \mathbf{0}& \mathbf{0}\\ \mathbf{0}& {\mathbf{I}\sigma }_{e2}^{2}& \mathbf{0}\\ \mathbf{0}& \mathbf{0}& {\mathbf{I}\sigma }_{e3}^{2}\end{array}\right) & \quad ={\mathbf{G}}_{A}+{\mathbf{G}}_{D}+\mathbf{R} \end{aligned}$$

Based on this notation, several equivalent estimators are derived in the following. We assume that variance components are known and that all individuals have a single record. Note that, by construction, we have a highly parameterized model, which in the GBLUP case has (many) more unknowns than records. I.e., each individual has an unknown additive and a dominance effect for each population but only one record in only one of the populations.

### SNP-BLUP

This model is parameterized in terms of SNP effects across the different populations, resulting in the following structure of the left-hand side (LHS) of the mixed model equations (MME) (for clarity, only the random effects part is shown):

$$\left(\begin{array}{ccccccc}\dots & & & & & & \\ & {\mathbf{Z}}_{1}^{\boldsymbol{^{\prime}}}{\mathbf{Z}}_{1}{\sigma }_{e1}^{-2}+\mathbf{I}{g}_{0a}^{11}& \mathbf{I}{g}_{0a}^{12}& \mathbf{I}{g}_{0a}^{13}& {\mathbf{Z}}_{1}^{\boldsymbol{^{\prime}}}{\mathbf{W}}_{1}{\sigma }_{e1}^{-2}& \mathbf{0}& \mathbf{0}\\ & \mathbf{I}{g}_{0a}^{21}& {\mathbf{Z}}_{2}^{\boldsymbol{^{\prime}}}{\mathbf{Z}}_{2}{\sigma }_{e2}^{-2}+\mathbf{I}{g}_{0a}^{22}& \mathbf{I}{g}_{0a}^{23}& \mathbf{0}& {\mathbf{Z}}_{2}^{\boldsymbol{^{\prime}}}{\mathbf{W}}_{2}{\sigma }_{e2}^{-2}& \mathbf{0}\\ & \mathbf{I}{g}_{0a}^{31}& \mathbf{I}{g}_{0a}^{32}& {\mathbf{Z}}_{3}^{\boldsymbol{^{\prime}}}{\mathbf{Z}}_{3}{\sigma }_{e3}^{-2}+\mathbf{I}{g}_{0a}^{33}& \mathbf{0}& \mathbf{0}& {\mathbf{Z}}_{3}^{\boldsymbol{^{\prime}}}{\mathbf{W}}_{3}{\sigma }_{e3}^{-2}\\ & {\mathbf{W}}_{1}^{\boldsymbol{^{\prime}}}{\mathbf{Z}}_{1}{\sigma }_{e1}^{-2}& \mathbf{0}& \mathbf{0}& {\mathbf{W}}_{1}^{^{\prime}}{\mathbf{W}}_{1}{\sigma }_{e1}^{-2}+\mathbf{I}{g}_{0d}^{11}& \mathbf{I}{g}_{0d}^{12}& \mathbf{I}{g}_{0d}^{13}\\ & \mathbf{0}& {\mathbf{W}}_{2}^{\boldsymbol{^{\prime}}}{\mathbf{Z}}_{2}{\sigma }_{e2}^{-2}& \mathbf{0}& \mathbf{I}{g}_{0d}^{21}& {\mathbf{W}}_{2}^{^{\prime}}{\mathbf{W}}_{2}{\sigma }_{e2}^{-2}+\mathbf{I}{g}_{0d}^{22}& \mathbf{I}{g}_{0d}^{23}\\ & \mathbf{0}& \mathbf{0}& {\mathbf{W}}_{3}^{\boldsymbol{^{\prime}}}{\mathbf{Z}}_{3}{\sigma }_{e3}^{-2}& \mathbf{I}{g}_{0d}^{31}& \mathbf{I}{g}_{0d}^{32}& {\mathbf{W}}_{3}^{^{\prime}}{\mathbf{W}}_{3}{\sigma }_{e3}^{-2}+\mathbf{I}{g}_{0d}^{33}\end{array}\right)$$

This LHS is composed of several dense blocks of the form ${\mathbf{Z}}_{1}^{\boldsymbol{^{\prime}}}{\mathbf{Z}}_{1}{\sigma }_{e1}^{-2}+\mathbf{I}{g}_{0a}^{11}$, several sparse blocks of the form $\mathbf{I}{g}_{0a}^{12}$, and several zero blocks. This makes the use of current “state of the art” sparse matrix software inefficient, because most gains due to sparsity are lost. As a result, it may be more efficient to work with full storage, i.e. dense matrices. However, the size of the equations is rather large, i.e. $6m$, where $m$ is the number of SNPs. For instance, an analysis with 50 K SNP panels would involve MME of size 300 K and the inversion of the LHS would consume ${O\left(6m\right)}^{3}$ operations. The size of these equations are, however, invariant to the number of records $n$.

### GBLUP using a standard multiple trait formulation

There are two possible implementations of GBLUP. The first considers three additive values and three dominance values per individual (one for each population). For each random effect, the MME use a single matrix that is arbitrarily scaled across all populations (the scale is arbitrary because there is no meaningful scaling factor that yields coherent “relationships” across the three populations). For instance, a possible matrix for the additive effect is:

$${\mathbf{G}}_{A}^{*}=\left(\begin{array}{ccc}{\mathbf{Z}}_{1}{\mathbf{Z}}_{1}^{\mathbf{^{\prime}}}& {\mathbf{Z}}_{1}{\mathbf{Z}}_{2}^{\mathbf{^{\prime}}}& {\mathbf{Z}}_{1}{\mathbf{Z}}_{3}^{\mathbf{^{\prime}}}\\ {\mathbf{Z}}_{2}{\mathbf{Z}}_{1}^{\mathbf{^{\prime}}}& {\mathbf{Z}}_{2}{\mathbf{Z}}_{2}^{\mathbf{^{\prime}}}& {\mathbf{Z}}_{2}{\mathbf{Z}}_{3}^{\mathbf{^{\prime}}}\\ {\mathbf{Z}}_{3}{\mathbf{Z}}_{1}^{\mathbf{^{\prime}}}& {\mathbf{Z}}_{3}{\mathbf{Z}}_{2}^{\mathbf{^{\prime}}}& {\mathbf{Z}}_{3}{\mathbf{Z}}_{3}^{\mathbf{^{\prime}}}\end{array}\right)$$

with $${\left({\mathbf{G}}_{A}^{*}\right)}^{-1}=\left(\begin{array}{ccc}{\mathbf{G}}_{A}^{*\left(11\right)}& {\mathbf{G}}_{A}^{*\left(12\right)}& {\mathbf{G}}_{A}^{*\left(13\right)}\\ {\mathbf{G}}_{A}^{*\left(21\right)}& {\mathbf{G}}_{A}^{*\left(22\right)}& {\mathbf{G}}_{A}^{*\left(23\right)}\\ {\mathbf{G}}_{A}^{*\left(31\right)}& {\mathbf{G}}_{A}^{*\left(32\right)}& {\mathbf{G}}_{A}^{*\left(33\right)}\end{array}\right)$$.

Note that the cost of inversion of ${\mathbf{G}}_{A}^{*}$ is $O\left({n}^{3}\right)$. Note also that, in order to describe covariances between individuals, ${\mathbf{G}}_{A}^{*}$ must be multiplied by the SNP effect variance (e.g. ${\sigma }_{a1}^{2}$), rather than by the population variance, which is why we prefer not to refer to ${\mathbf{G}}_{A}^{*}$ as containing “relationships”. A similar matrix ${\mathbf{G}}_{D}^{*}$ is defined for dominance effects. The two matrices ${\mathbf{G}}_{A}^{*}$ and ${\mathbf{G}}_{D}^{*}$ are used in a multiple trait formulation with missing records for each trait, using the Kronecker factorization. This results in the following structure of LHS, as for multiple trait analysis (for clarity, only a portion of the structure is shown, for additive effects for two of the three populations):

$$\left(\begin{array}{cccccccc}\dots & & & & & & & \\ & \mathbf{I}{\sigma }_{e1}^{-2}+{\mathbf{G}}_{A}^{*\left(11\right)}{g}_{0a}^{11}& {\mathbf{G}}_{A}^{*\left(12\right)}{g}_{0a}^{11}& {\mathbf{G}}_{A}^{*\left(13\right)}{g}_{0a}^{11}& {\mathbf{G}}_{A}^{*\left(11\right)}{g}_{0a}^{12}& {\mathbf{G}}_{A}^{*\left(12\right)}{g}_{0a}^{12}& {\mathbf{G}}_{A}^{*\left(13\right)}{g}_{0a}^{12}& \\ & {\mathbf{G}}_{A}^{*\left(21\right)}{g}_{0a}^{11}& {\mathbf{G}}_{A}^{*\left(22\right)}{g}_{0a}^{11}& {\mathbf{G}}_{A}^{*\left(23\right)}{g}_{0a}^{11}& {\mathbf{G}}_{A}^{*\left(21\right)}{g}_{0a}^{12}& {\mathbf{G}}_{A}^{*\left(22\right)}{g}_{0a}^{12}& {\mathbf{G}}_{A}^{*\left(23\right)}{g}_{0a}^{12}& \\ & {\mathbf{G}}_{A}^{*\left(31\right)}{g}_{0a}^{11}& {\mathbf{G}}_{A}^{*\left(32\right)}{g}_{0a}^{11}& {\mathbf{G}}_{A}^{*\left(33\right)}{g}_{0a}^{11}& {\mathbf{G}}_{A}^{*\left(31\right)}{g}_{0a}^{12}& {\mathbf{G}}_{A}^{*\left(32\right)}{g}_{0a}^{12}& {\mathbf{G}}_{A}^{*\left(33\right)}{g}_{0a}^{12}& \\ & {\mathbf{G}}_{A}^{*\left(11\right)}{g}_{0a}^{21}& {\mathbf{G}}_{A}^{*\left(12\right)}{g}_{0a}^{21}& {\mathbf{G}}_{A}^{*\left(13\right)}{g}_{0a}^{21}& {\mathbf{G}}_{A}^{*\left(11\right)}{g}_{0a}^{22}& {\mathbf{G}}_{A}^{*\left(12\right)}{g}_{0a}^{22}& {\mathbf{G}}_{A}^{*\left(13\right)}{g}_{0a}^{22}& \\ & {\mathbf{G}}_{A}^{*\left(21\right)}{g}_{0a}^{21}& {\mathbf{G}}_{A}^{*\left(22\right)}{g}_{0a}^{21}& {\mathbf{G}}_{A}^{*\left(23\right)}{g}_{0a}^{21}& {\mathbf{G}}_{A}^{*\left(12\right)}{g}_{0a}^{21}& \mathbf{I}{\sigma }_{e2}^{-2}+{\mathbf{G}}_{A}^{*\left(22\right)}{g}_{0a}^{22}& {\mathbf{G}}_{A}^{*\left(23\right)}{g}_{0a}^{22}& \\ & {\mathbf{G}}_{A}^{*\left(31\right)}{g}_{0a}^{21}& {\mathbf{G}}_{A}^{*\left(32\right)}{g}_{0a}^{21}& {\mathbf{G}}_{A}^{*\left(33\right)}{g}_{0a}^{21}& {\mathbf{G}}_{A}^{*\left(13\right)}{g}_{0a}^{21}& {\mathbf{G}}_{A}^{*\left(23\right)}{g}_{0a}^{22}& {\mathbf{G}}_{A}^{*\left(33\right)}{g}_{0a}^{22}& \\ & & & & & & & \dots \end{array}\right).$$

A similar pattern results for the dominance effects. There are also corresponding cross-products of incidence matrices in the additive x dominance blocks, e.g. $\mathbf{I}{\sigma }_{e1}^{-2}$. Thus, the LHS are composed of a dense formulation where each row has two times three blocks of size $n$, leading to $6n$ equations (and inversion cost of $O{\left(6n\right)}^{3}$). To our knowledge, this is the formulation used by standard BLUP/REML software programs (blupf90, Wombat, ASREML) with elements stored in memory. Note that these MME require ${\mathbf{G}}_{{\varvec{A}}}$ and ${\mathbf{G}}_{{\varvec{D}}}$ to be full rank, which is often not the case (because of the presence of clones, or as a result of “centering” the $\mathbf{Z}$ and $\mathbf{W}$ matrices, or because $n>m$). Matrices ${\mathbf{G}}_{{\varvec{A}}}$ and ${\mathbf{G}}_{{\varvec{D}}}$ may, therefore, require some “blending”.

### GBLUP using a compact formulation

The second option for the formulation of GBLUP, which requires much less memory, directly uses the inverses of the covariance matrices ${\mathbf{G}}_{{\varvec{A}}}$ and ${\mathbf{G}}_{{\varvec{D}}}$ (again, assuming that they are, or have been made, invertible), rather than the Kronecker factorization of covariances, e.g.:

$${\mathbf{G}}_{{\varvec{A}}}^{-1}={\left(\begin{array}{ccc}{{\mathbf{Z}}_{1}\sigma }_{a1}^{2}{\mathbf{Z}}_{1}^{\mathrm{^{\prime}}} & {\mathbf{Z}}_{1}{\sigma }_{a12}{\mathbf{Z}}_{2}^{\mathrm{^{\prime}}}& {\mathbf{Z}}_{1}{\sigma }_{a13}{\mathbf{Z}}_{3}^{\mathrm{^{\prime}}}\\ {\mathbf{Z}}_{2}{\sigma }_{a21}{\mathbf{Z}}_{1}^{\mathrm{^{\prime}}}& {{\mathbf{Z}}_{2}\sigma }_{a2}^{2}{\mathbf{Z}}_{2}^{\mathrm{^{\prime}}}& {\mathbf{Z}}_{2}{\sigma }_{a23}{\mathbf{Z}}_{3}^{\mathrm{^{\prime}}}\\ {\mathbf{Z}}_{3}{\sigma }_{a31}{\mathbf{Z}}_{1}^{\mathrm{^{\prime}}}& {\mathbf{Z}}_{3}{\sigma }_{a32}{\mathbf{Z}}_{2}^{\mathrm{^{\prime}}}& {{\mathbf{Z}}_{3}\sigma }_{a3}^{2}{\mathbf{Z}}_{3}^{\mathrm{^{\prime}}}\end{array}\right)}^{-1}=\left(\begin{array}{ccc}{\mathbf{G}}_{A}^{\left(11\right)}& {\mathbf{G}}_{A}^{\left(12\right)}& {\mathbf{G}}_{A}^{\left(13\right)}\\ {\mathbf{G}}_{A}^{\left(21\right)}& {\mathbf{G}}_{A}^{\left(22\right)}& {\mathbf{G}}_{A}^{\left(23\right)}\\ {\mathbf{G}}_{A}^{\left(31\right)}& {\mathbf{G}}_{A}^{\left(32\right)}& {\mathbf{G}}_{A}^{\left(33\right)}\end{array}\right),$$

and then it uses the following LHS structure:

$$\left(\begin{array}{ccccccc}\dots & & & & & & \\ & \mathbf{I}{\sigma }_{e1}^{-2}+{\mathbf{G}}_{A}^{\left(11\right)}& {\mathbf{G}}_{A}^{\left(12\right)}& {\mathbf{G}}_{A}^{\left(13\right)}& \mathbf{I}{\sigma }_{e1}^{-2}& \mathbf{0}& \mathbf{0}\\ & {\mathbf{G}}_{A}^{\left(21\right)}& \mathbf{I}{\sigma }_{e2}^{-2}+{\mathbf{G}}_{A}^{\left(22\right)}& {\mathbf{G}}_{A}^{\left(23\right)}& \mathbf{0}& \mathbf{I}{\sigma }_{e2}^{-2}& \mathbf{0}\\ & {\mathbf{G}}_{A}^{\left(31\right)}& {\mathbf{G}}_{A}^{\left(32\right)}& {\mathbf{I}{\sigma }_{e3}^{-2}+\mathbf{G}}_{A}^{\left(33\right)}& \mathbf{0}& \mathbf{0}& \mathbf{I}{\sigma }_{e3}^{-2}\\ & \mathbf{I}{\sigma }_{e1}^{-2}& \mathbf{0}& \mathbf{0}& \mathbf{I}{\sigma }_{e1}^{-2}+{\mathbf{G}}_{D}^{\left(11\right)}& {\mathbf{G}}_{D}^{\left(12\right)}& {\mathbf{G}}_{D}^{\left(13\right)}\\ & \mathbf{0}& \mathbf{I}{\sigma }_{e2}^{-2}& \mathbf{0}& {\mathbf{G}}_{D}^{\left(21\right)}& \mathbf{I}{\sigma }_{e2}^{-2}+{\mathbf{G}}_{D}^{\left(22\right)}& {\mathbf{G}}_{D}^{\left(23\right)}\\ & \mathbf{0}& \mathbf{0}& \mathbf{I}{\sigma }_{e3}^{-2}& {\mathbf{G}}_{D}^{\left(31\right)}& {\mathbf{G}}_{D}^{\left(32\right)}& \mathbf{I}{\sigma }_{e3}^{-2}+{\mathbf{G}}_{D}^{\left(33\right)}\end{array}\right),$$

which is of size $2n$, but is still considerably dense.

None of the above alternatives (SNP-BLUP or either GBLUP) are very satisfying since they all involve large matrices of size $>n$ or $>m$, and only SNP-BLUP directly leads to estimates of SNP effects, which are required to predict newly genotyped animals (without re-running the evaluation) or to arrange assortative matings.

### GLS and selection index formulation

An alternative is to use GLS followed by SI theory, as shown by Henderson [3, 4]. First, we estimate the fixed effects by GLS:

$$\widehat{{\varvec{\upbeta}}}={\left({\mathbf{X}}^{\mathrm{^{\prime}}}{\mathbf{V}}^{-1}\mathbf{X}\right)}^{-1}{\mathbf{X}}^{\mathrm{^{\prime}}}{\mathbf{V}}^{-1}\mathbf{y},$$

after which SNP effects are estimated through covariances as:

$$\widehat{\mathbf{a}}={\mathbf{C}}_{a}^{\mathrm{^{\prime}}}{\mathbf{V}}^{-1}\left(\mathbf{y}-\mathbf{X}\widehat{{\varvec{\upbeta}}}\right),$$

$$\widehat{\mathbf{d}}={\mathbf{C}}_{\mathrm{d}}^{\mathbf{^{\prime}}}{\mathbf{V}}^{-1}\left(\mathbf{y}-\mathbf{X}\widehat{{\varvec{\upbeta}}}\right).$$

where ${\mathbf{C}}_{a}=Cov\left(\mathbf{y},\mathbf{a}{^{\prime}}\right)$ and ${\mathbf{C}}_{d}=Cov\left(\mathbf{y},{\mathbf{d}}^{\mathrm{^{\prime}}}\right)$. Constructing $\mathbf{V}$ is actually easy, because it is just a sum of matrices. Provided $\mathbf{V}$ is invertible, and using a single inversion, we can solve for ${\varvec{\upbeta}}$ first and then backsolve for $\mathbf{a}$ and $\mathbf{d}$. In the multi-population case, this may be computationally simpler than the SNP-BLUP and GBLUP strategies because $\mathbf{V}$ is smaller (of size $n$) than several of the $\mathbf{G}$ matrices or LHS matrices in the SNP-BLUP and GBLUP formulations. Moreover, $\mathbf{V}$ is invertible by construction, while ${\mathbf{G}}_{{\varvec{A}}}$ and ${\mathbf{G}}_{{\varvec{D}}}$ may not be full rank and may need some “blending”. Several authors [8–10] have already pointed out that the GLS formulation is computationally more compact when MME are non-sparse and requires, in principle, fewer computations.

Therefore, to estimate SNP effects for multi-population evaluation, we propose the following algorithm based on GLS and SI. Assume that there are ${n}_{1}$, ${n}_{2}$, and ${n}_{3}$ records for each population:

1. Read data, build $\mathbf{X}$, $\mathbf{Z}$ and $\mathbf{W}$.
2. Create empty $\mathbf{V}$ of the right size ($n={n}_{1}+{n}_{2}+{n}_{3}$).
3. Add residual variances to $\mathbf{V}$.
4. Add contributions from populations 1, 2, 3 and $a$, $d$, $e$ to $\mathbf{V}$.
5. Invert $\mathbf{V}$ (with associated cost $O({n}^{3})$).
6. Solve for fixed effects: $\widehat{{\varvec{\upbeta}}}={\left({\mathbf{X}}^{\mathbf{^{\prime}}}{\mathbf{V}}^{-1}\mathbf{X}\right)}^{-1}{\mathbf{X}}^{\mathbf{^{\prime}}}{\mathbf{V}}^{-1}\mathbf{y}$.
7. Solve for random effects using covariances as described in the following. This can be done in steps (${\mathbf{Z}}_{1}$, then ${\mathbf{Z}}_{2}$, etc.), specifically:
  $$\widehat{\mathbf{a}}={\mathbf{C}}_{\mathrm{a}}^{\mathbf{^{\prime}}}{\mathbf{V}}^{-1}\left(\mathbf{y}-\mathbf{X}\widehat{{\varvec{\upbeta}}}\right)$$
  $$\widehat{\mathbf{d}}={\mathbf{C}}_{\mathrm{d}}^{\mathbf{^{\prime}}}{\mathbf{V}}^{-1}\left(\mathbf{y}-\mathbf{X}\widehat{{\varvec{\upbeta}}}\right).$$

To compute $\widehat{\mathbf{a}}$ we need ${\mathbf{C}}_{\mathrm{a}}^{\mathbf{^{\prime}}}=\mathrm{Cov}\left(\mathbf{a},{\mathbf{y}}^{\mathbf{^{\prime}}}\right)$ the covariance of $\mathbf{a}$ with $\mathbf{y}$, which is:

$${\mathbf{C}}_{a}^{\mathbf{^{\prime}}}=\left(\begin{array}{ccc}{\mathbf{I}\sigma }_{a1}^{2}& \mathbf{I}{\sigma }_{a12}& \mathbf{I}{\sigma }_{a13}\\ \mathbf{I}{\sigma }_{a21}& {\mathbf{I}\sigma }_{a2}^{2}& \mathbf{I}{\sigma }_{a23}\\ \mathbf{I}{\sigma }_{a31}& \mathbf{I}{\sigma }_{a32}& {\mathbf{I}\sigma }_{a3}^{2}\end{array}\right)\left(\begin{array}{ccc}{\mathbf{Z}}_{1}^{\mathbf{^{\prime}}}& \mathbf{0}& \mathbf{0}\\ \mathbf{0}& {\mathbf{Z}}_{2}^{\mathbf{^{\prime}}}& \mathbf{0}\\ \mathbf{0}& \mathbf{0}& {\mathbf{Z}}_{3}^{\mathbf{^{\prime}}}\end{array}\right)=\left(\begin{array}{ccc}{\mathbf{Z}}_{1}^{\mathbf{^{\prime}}}{\sigma }_{a1}^{2}& {\mathbf{Z}}_{2}^{\mathbf{^{\prime}}}{\sigma }_{a12}& {\mathbf{Z}}_{3}^{\mathbf{^{\prime}}}{\sigma }_{a13}\\ {\mathbf{Z}}_{1}^{\mathbf{^{\prime}}}{\sigma }_{a21}& {{\mathbf{Z}}_{2}^{\mathbf{^{\prime}}}\sigma }_{a2}^{2}& {\mathbf{Z}}_{3}^{\mathbf{^{\prime}}}{\sigma }_{a23}\\ {\mathbf{Z}}_{1}^{\mathbf{^{\prime}}}{\sigma }_{a31}& {\mathbf{Z}}_{2}^{\mathbf{^{\prime}}}{\sigma }_{a32}& {{\mathbf{Z}}_{3}^{\mathbf{^{\prime}}}\sigma }_{a3}^{2}\end{array}\right).$$

The algorithm to compute $\widehat{\mathbf{a}}$ then is:

- Build $${\mathbf{y}}^{\mathrm{c}}={\mathbf{V}}^{-1}\left(\mathbf{y}-\mathbf{X}\widehat{{\varvec{\upbeta}}}\right)$$.
- Then $$\mathbf{v}=\left(\begin{array}{c}{\mathbf{v}}_{1}\\ {\mathbf{v}}_{2}\\ {\mathbf{v}}_{3}\end{array}\right)=\left(\begin{array}{ccc}{\mathbf{Z}}_{1}^{\mathbf{^{\prime}}}& \mathbf{0}& \mathbf{0}\\ \mathbf{0}& {\mathbf{Z}}_{2}^{\mathbf{^{\prime}}}& \mathbf{0}\\ \mathbf{0}& \mathbf{0}& {\mathbf{Z}}_{3}^{\mathbf{^{\prime}}}\end{array}\right)\left(\begin{array}{c}{\mathbf{y}}_{1}^{\mathrm{c}}\\ {\mathbf{y}}_{2}^{\mathrm{c}}\\ {\mathbf{y}}_{3}^{\mathrm{c}}\end{array}\right)=\left(\begin{array}{c}{\mathbf{Z}}_{1}^{\mathbf{^{\prime}}}{\mathbf{y}}_{1}^{\mathrm{c}}\\ {\mathbf{Z}}_{2}^{\mathbf{^{\prime}}}{\mathbf{y}}_{2}^{\mathrm{c}}\\ {{\mathbf{Z}}_{3}^{\mathbf{^{\prime}}}\mathbf{y}}_{3}^{\mathrm{c}}\end{array}\right)$$.

And finally $$\left(\begin{array}{c}{\widehat{\mathbf{a}}}_{1}\\ {\widehat{\mathbf{a}}}_{2}\\ {\widehat{{\varvec{a}}}}_{3}\end{array}\right)=\left(\begin{array}{ccc}{\mathbf{I}\sigma }_{a1}^{2}\boldsymbol{ }& \mathbf{I}{\sigma }_{a12}& \mathbf{I}{\sigma }_{a13}\\ \mathbf{I}{\sigma }_{a21}& {\mathbf{I}\sigma }_{a2}^{2}& \mathbf{I}{\sigma }_{a23}\\ \mathbf{I}{\sigma }_{a31}& \mathbf{I}{\sigma }_{a32}& {\mathbf{I}\sigma }_{a3}^{2}\end{array}\right)\left(\begin{array}{c}{\mathbf{v}}_{1}\\ {\mathbf{v}}_{2}\\ {\mathbf{v}}_{3}\end{array}\right)=\left(\begin{array}{c}{{\mathbf{v}}_{1}\sigma }_{a1}^{2}+{\mathbf{v}}_{2}{\sigma }_{a12}+{\mathbf{v}}_{3}{\sigma }_{a13}\\ {\mathbf{v}}_{1}{\sigma }_{a21}+{\mathbf{v}}_{2}{\sigma }_{a2}^{2}+{\mathbf{v}}_{3}{\sigma }_{a23}\\ {\mathbf{v}}_{1}{\sigma }_{a31}+{\mathbf{v}}_{2}{\sigma }_{a32}+{\mathbf{v}}_{3}{\sigma }_{a3}\end{array}\right).$$

We then proceed similarly for $\mathbf{d}$.

Useful by-products of the inversion-based (as opposed to iterative) computation of BLUP are reliabilities random effect predictions, which are usually obtained from prediction error variances. Prediction error variances of estimates of SNP effects can also be used in genome-wide association studies to assess significance of SNP effects. For the particular case of GLS + SI, individual reliabilities can be obtained from the inverses ${\mathbf{V}}^{-1}$ and ${\left({\mathbf{X}}^{\mathbf{^{\prime}}}{\mathbf{V}}^{-1}\mathbf{X}\right)}^{-1}$ and from the covariances of $\mathbf{y}$ with the different random effects [4]. Efficient algorithms for REML based on the GLS formulation also exist [10, 11].

### Iterative methods for genetic evaluation

In contrast to the exact inversion methods described above, most genetic evaluation software used for livestock use iterative methods that do not invert matrices or even set up MME explicitly, e.g. [12]. Iterative methods have two inconveniences compared to exact inversion methods: (1) convergence may be slow and is a priori unpredictable, and (2) other information such as reliabilities from the inverses of the MME is lost. For the types of multi-population models considered here, convergence of iterative methods is not always good, because there are many more effects than records and, for a given number of records, the condition number of the MME worsens with each extra effect.

We are not aware of iterative methods that use the GLS + SI formulation. However, in principle, it is possible to solve $\left({\mathbf{X}}^{\mathbf{^{\prime}}}{\mathbf{V}}^{-1}\mathbf{X}\right)\widehat{{\varvec{\upbeta}}}= {\mathbf{X}}^{\mathbf{^{\prime}}}{\mathbf{V}}^{-1}\mathbf{y}$ without inversion or even storage of matrices, by first solving $\mathbf{V}{\varvec{\Theta}}=\mathbf{y}$ (so ${\varvec{\Theta}}={\mathbf{V}}^{-1}\mathbf{y})$ and $\mathbf{V}\mathbf{M}=\mathbf{X}$ (so $\mathbf{M}={\mathbf{V}}^{-1}\mathbf{X})$, and then $\left({\mathbf{X}}^{\mathbf{^{\prime}}}\mathbf{M}\right)\widehat{{\varvec{\upbeta}}}={\mathbf{X}}^{\mathbf{^{\prime}}}{\varvec{\Theta}}$. To estimate SNP effects using SI, it is useful to note that ${\mathbf{y}}^{c}={\varvec{\Theta}}-\mathbf{M}\widehat{{\varvec{\upbeta}}}$.

## Discussion

Henderson’s formulation of MME allowed the use of linear methods for genetic evaluation as opposed to, say, likelihoods in pedigrees [13]. The two key discoveries of the MME and of the fast (and sparse) construction of the inverse of the pedigree-based numerator relationship matrix led to computational efficiency, not only for estimation of breeding values, but also for estimation of variance components, in which most algorithms use predictions of random effects and elements from the inverse of the MME.

However, sparsity of the MME is only partly retained when using dense marker genotypes, as these invariably lead to dense cross-products, either as incidence matrices ($\mathbf{Z}\mathbf{^{\prime}}\mathbf{Z}$) or as covariance matrices $(\mathbf{Z}{\mathbf{Z}}^{\mathbf{^{\prime}}}$). In addition, the latter $(\mathbf{Z}{\mathbf{Z}}^{\mathbf{^{\prime}}}$) need to be inverted for their inclusion in MME. Within the framework of the use of linear models for genetic evaluation, the use of any computing strategy, including GBLUP, SNP-BLUP, and GLS + SI, is now mostly a matter of convenience for the user (availability of software) and for the programmer (general and/or easy formulations are preferred to complex ones). Computationally, the efficiency of the approach depends on the number of records and on the number of markers. In our particular problem of multi-breed prediction, generally, SNP-BLUP is computationally easier when $n>m$ (more records than markers), GLS + SI is easier when $m>n$ (more markers than records), and GBLUP is easier when $m>n$ and the model is notoriously complex to fit (e.g. random regressions on time or temperature, correlated animal effects, etc.).

SNP-BLUP models are interesting because they yield estimates of marker effects. These allow so-called “indirect” predictions (e.g. for young genotyped animals with no own record) to be calculated as the sum of SNP effect estimates weighted by gene content at SNPs. Dominance (or higher order interaction) effect estimates allow matings that maximize performance to be optimized. GBLUP or GLS + SI also allow estimates of marker effects to be obtained using covariances as explained before.

Compared to SNP-BLUP and GBLUP, an advantage of GLS + SI is the ability to fit increasingly complex models without increasing the dimensions of the GLS. Examples are genotype-by-genotype and genotype-by-environment interactions [14, 15], which are of interest, respectively, for planned matings and breeding for target environmental conditions.

The focus of this note was medium-sized populations of up to ~ 100K individuals and/or genotyped markers. In these populations, it is possible to fit rather complex models while maintaining the favorable features of exact methods, including shorter computing time, computed reliabilities, maximum likelihood algorithms, and even genome-wide associations studies. For very large data sets, iteration on data [12, 16–19] are good options, as they do not require cross-products to be explicitly computed (or only partially) or inversion of the MME.

## Conclusions

We have shown that for multi-breed prediction, Henderson’s MME (either in terms of marker effects or of individual animal effects—SNP-BLUP and GBLUP, respectively) do not necessarily lead to the most computationally efficient approach, although it is a very flexible one. If most individuals are genotyped, then other more parsimonious alternatives could be considered in addition to GBLUP or SNP-BLUP. The use of GLS combined with SI is one of these.

## Data Availability

A program that implements SNP-BLUP and GLS followed by selection index is available at https://genoweb.toulouse.inra.fr/~alegarra/test.r.
